# Cell Stress Induced Stressome Release Including Damaged Membrane Vesicles and Extracellular HSP90 by Prostate Cancer Cells

**DOI:** 10.3390/cells9030755

**Published:** 2020-03-19

**Authors:** Takanori Eguchi, Chiharu Sogawa, Kisho Ono, Masaki Matsumoto, Manh Tien Tran, Yuka Okusha, Benjamin J. Lang, Kuniaki Okamoto, Stuart K. Calderwood

**Affiliations:** 1 Department of Dental Pharmacology, Graduate School of Medicine, Dentistry and Pharmaceutical Sciences, Okayama University, Okayama 700-8525, Japan; caoki@md.okayama-u.ac.jp (C.S.); trantienmanh1508@gmail.com (M.T.T.); yokusha@bidmc.harvard.edu (Y.O.); k-oka@okayama-u.ac.jp (K.O.); 2 Advanced Research Center for Oral and Craniofacial Sciences, Graduate School of Medicine, Dentistry and Pharmaceutical Sciences, Okayama University, Okayama 700-8525, Japan; 3 Department of Oral and Maxillofacial Surgery, Okayama University Hospital, Okayama 700-0914, Japan; de20012@s.okayama-u.ac.jp; 4 Department of Molecular and Cellular Biology, Medical Institute of Bioregulation, Kyushu University, Fukuoka 812-8582, Japan; masakim@bioreg.kyushu-u.ac.jp; 5 Department of Radiation Oncology, Beth Israel Deaconess Medical Center, Harvard Medical School, Boston, MA 02115, USA; bjlang@bidmc.harvard.edu

**Keywords:** cell stress response, stressome, extracellular vesicle, heat shock protein 90 (HSP90), cell division control 37 (CDC37), prostate cancer, exosome, ectosome

## Abstract

Tumor cells exhibit therapeutic stress resistance-associated secretory phenotype involving extracellular vesicles (EVs) such as oncosomes and heat shock proteins (HSPs). Such a secretory phenotype occurs in response to cell stress and cancer therapeutics. HSPs are stress-responsive molecular chaperones promoting proper protein folding, while also being released from cells with EVs as well as a soluble form known as alarmins. We have here investigated the secretory phenotype of castration-resistant prostate cancer (CRPC) cells using proteome analysis. We have also examined the roles of the key co-chaperone CDC37 in the release of EV proteins including CD9 and epithelial-to-mesenchymal transition (EMT), a key event in tumor progression. EVs derived from CRPC cells promoted EMT in normal prostate epithelial cells. Some HSP family members and their potential receptor CD91/LRP1 were enriched at high levels in CRPC cell-derived EVs among over 700 other protein types found by mass spectrometry. The small EVs (30–200 nm in size) were released even in a non-heated condition from the prostate cancer cells, whereas the EMT-coupled release of EVs (200–500 nm) and damaged membrane vesicles with associated HSP90α was increased after heat shock stress (HSS). GAPDH and lactate dehydrogenase, a marker of membrane leakage/damage, were also found in conditioned media upon HSS. During this stress response, the intracellular chaperone CDC37 was transcriptionally induced by heat shock factor 1 (HSF1), which activated the CDC37 core promoter, containing an interspecies conserved heat shock element. In contrast, knockdown of CDC37 decreased EMT-coupled release of CD9-containing vesicles. Triple siRNA targeting CDC37, HSP90α, and HSP90β was required for efficient reduction of this chaperone trio and to reduce tumorigenicity of the CRPC cells in vivo. Taken together, we define “stressome” as cellular stress-induced all secretion products, including EVs (200–500 nm), membrane-damaged vesicles and remnants, and extracellular HSP90 and GAPDH. Our data also indicated that CDC37 is crucial for the release of vesicular proteins and tumor progression in prostate cancer.

## 1. Introduction

Tumor cells are often exposed to various stresses such as immune/inflammatory stress, therapeutics [1], hypoxia [2,3,4], acidification, oxidative stress [5,6], starvation [7], nutrient stress [8], heat and cold [9,10], thermal stress, replication stress [11], endoplasmic reticulum (ER) stress, neurotoxic stress [12], genotoxic (DNA damage) [13] and proteotoxic stress [14,15]. Heat shock proteins (HSPs) were originally found to be induced upon heat shock stress (HSS) [9]. Later studies have revealed that other types of stresses can also induce HSPs, including hypoxia [16] and nutrient starvation [7]. HSPs are molecular chaperones that assist in proper protein folding and re-folding in the cells, playing stress-resistant roles in anti-apoptotic activity [9] against radiation therapy, chemotherapy, and immunotherapy. It has been shown that HSPs are often increased in tumor cells and are involved in the properties of tumor progression such as increased migration, invasion, and metastasis [17,18]. Additionally, extracellular HSPs are released from cells with vesicles as well as a soluble form [4,19,20]. Notably, HSPs and vesicles were co-released upon cell stress and cell damage such as molecular targeted therapeutic stress [21,22], anticancer therapeutic DNA damage stress [23,24], and HSS [25,26]. Extracellular HSPs are also known as alarmin or damage-/danger-associated molecular patterns (DAMP) that are released from cells upon tissue damage [27,28]. 

Extracellular vesicle (EV) is the generic term for particles naturally released from the cell that are delimited by lipid bilayer and cannot replicate, i.e., do not contain a functional nucleus [29]. EVs often contain a variety of molecular cargos such as proteins, small and large RNAs, DNA, lipid, glycans, mineral such as calcium, and metabolites that are secreted by cells [30,31,32,33]. Major EV subtypes are endosome-origin “exosomes” and plasma membrane-derived “ectosomes” (also known as microparticles or microvesicles) [34,35]. Authors have been urged to consider use of operational terms for EV subtypes that refer to physical characteristics of EVs, such as size; small EVs (sEV) and medium/large EVs (m/lEVs), with ranges defined, for example, < 200 nm (small) or > 200 nm (large and/or medium), respectively [29,36,37]. Additionally, according to the history of discoveries and characteristics of EVs, additional types of EVs have been reported consisting of oncosomes (named after oncogenic EVs) [38,39], large oncosomes (1–10 μm) [40], matrix vesicles [41], migrasomes [42], exopheres (≈4 μm), exomeres (≈35 nm), and bacterial outer membrane vesicles (OMV) [43]. These vesicles often play basic roles in discarding molecules unfavorable for cells [44], while also mediating intercellular communication by transferring their cargos to recipient cells or organs in local and/or distant tissues [45]. Among various concepts of EVs, oncosomes have been shown to promote processes in tumor progression such as epithelial-to-mesenchymal transition (EMT) by transferring oncogenic molecules [46,47,48]. In addition, the enhancement of EMT properties was often coupled with increased release of EVs [22,49]. These facts prompted us to make a concept of EMT-coupled vesicle release.

Among many molecular cargos, HSPs are major signatures often found within sEV such as exosomes, ectosomes, and oncosomes [4,19,20,27]. According to the minimal information for studies of extracellular vesicles 2018 (MISEV2018), EV markers are categorized into five categories and the category 2 (cytosolic proteins recovered in EVs) includes heat shock cognate 70 (HSC70/HSPA8: essential for autophagy), HSP70, and HSP90β (HSP90AB1/HSP84), while category 4 (transmembrane, lipid-bound, and soluble proteins associated to other intracellular compartments than plasma membrane/endosomes) includes ER chaperones such as Grp94/HSP90B and BiP/HSPA5 [29]. Moreover, sEV derived from oral cancer cells contained a variety of HSPs, including HSP90α (HSP90AA1), HSP90β, TRAP1 (mitochondrial HSP90), HSP105, HSP70 (HSP72/HSPA1A and HSP70B’/HSPA6), GRP78/HSPA5 (ER chaperone), and HSC70 [20]. Among these HSPs, two HSP90 isoforms (HSP90α and HSP90β) were enriched in sEV derived from high metastatic cancer cells compared to low metastatic ones [20]. The high expression levels of HSP90α/β are correlated with poor prognosis in patients suffering from head and neck cancers [20]. Several studies, including ours, have indicated that HSP90 is essential in stress resistance in cancer cells [19,50,51,52]. Cell division control 37 (CDC37), a key co-chaperone for HSP90, is highly expressed in castration-resistant prostate cancer (CRPC) cells as compared to normal prostate epithelial cells or prostate adenocarcinoma cells [4,53]. However, less is known regarding stress-responsive co-release of EVs and HSP90 from the resistant cells. We have therefore addressed this deficiency in this study. 

The growth of prostate cancers is often androgen-dependent, requiring factors such as testosterone and dihydrotestosterone (DHT) [54]. Therefore, androgen depletion therapy (ADT), also called hormone therapy, is often effective in prostate cancers. However, some types of prostate cancer are resistant to ADT and known as CRPC, a prostatic type of neuroendocrine tumors (NET), and this is also a phenotype found in aggressive pancreatic cancer [55,56,57]. Notably, tumorigenicity and EMT are key properties for recurrence and metastasis in such aggressive, resistant cancers [58,59]. In the androgen insensitivity, intracellular kinase signaling pathways are given higher priorities required for tumor progression. Indeed, CDC37, the protein kinase-specialized (kinome) co-chaperone of HSP90, was highly expressed in the CRPC cells as compared to the prostate adenocarcinoma cells and normal prostate epithelial cells [60]. CDC37 plays a fundamental role in chaperoning almost all members of the protein kinase family and participates in cancer by maintaining the activity of protein kinases involved in cell proliferation and transformation [61,62]. These include tyrosine kinases such as Src [63], and serine/threonine kinases in the Raf-ERK pathway [64], Akt, the inhibitor of NF-κB kinase (IKK) [65], and cyclin-dependent kinase 4 (CDK4) [66,67]. CDC37 functions primarily in a complex with HSP90 to mediate the three-dimensional (3D) folding and structural integrity of client proteins kinases [63,68]. CDC37 is particularly significant in prostate cancer as its overexpression leads to spontaneous prostate carcinogenesis in transgenic mice [69]. It has also been suggested that the high levels of oncogenic proteins present in most cancers make them dependent on molecular chaperones, a state referred to as “chaperone addiction” [61]. Thus, because of their large protein clienteles, the CDC37-HSP90 axis offers a critical target for inactivating multiple oncogenic pathways. Consequently, the inhibition of HSP90 in cancer is currently a major area of research [70,71]. However, less is known regarding the role of CDC37 in EV release and we have addressed this deficiency in this study.

In the present study, we therefore aimed (i) to reveal proteome signatures of EVs derived from CRPC cells, and (ii) to unveil stress-responsive vesicle release potentially coupled with resistant tumorigenicity in cancer.

## 2. Materials and Methods

### 2.1. Cell Culture

PC-3, a CRPC cell line, DU-145, a prostate adenocarcinoma cell line, and PNT2, an immortalized prostate epithelial cell line, were maintained in RPMI1640 medium with 5% to 10% FBS as described previously [4,60,72,73]. RWPE1, a human normal prostate cell line, was maintained in keratinocyte serum-free medium (ThermoFisher, Waltham, MA, USA) supplemented with recombinant human epidermal growth factor (hEGF) and bovine pituitary extract. Human prostate epithelial cells (PrEC) were maintained in PrEBM basal medium supplemented with bovine pituitary extract, triiodothyronine, insulin, hEGF, hydrocortisone, transferrin, epinephrine, gentamicin sulfate-amphotericin, and retinoic acid (Lonza, Basel, Switzerland).

### 2.2. Heat Shock Stress

For HSS, the medium was replaced to incubated medium at 43 or 37 °C and then put in a water bath at 43 or 37 °C, as described previously [25,74]. After HSS, cells were washed with PBS (-) and cultured in serum-free media. Cellular photomicrographs were taken at 24 h after medium replacement by using Floid Cell Imaging Station (ThermoFisher).

### 2.3. Extracellular Vesicle and Non-EV Fractions

For HSS experiments, after the above-mentioned HS, cells were washed with PBS and cultured in serum-free media for 24 h and then conditioned media was collected. At the time of harvest, the numbers of cells were counted using Countess (ThermoFisher, Waltham, MA, USA) and whole cell lysate was prepared as described below. Otherwise, cultured cells were washed with Hanks’ balanced salt solution (HBSS), and then further cultured in serum-free medium for 1 or 2 days. EV fraction was prepared using a modified polymer-based precipitation (PBP) method. Briefly, the conditioned medium was centrifuged at 2000× *g* for 30 min at 4 °C to remove cell debris. For studies of knockdown and EMT, the supernatant was filtered with a 0.2-µm syringe filter. Otherwise, the filter was not used. The supernatant was collected and centrifuged at 10,000× *g* for 30 min at 4 °C. The supernatant was collected and applied to an Amicon Ultra-15 Centrifugal Filter Device MW.100k (Merck, Kenilworth, NJ, USA) to concentrate the pre-EV fraction to less than 1 mL and to separate non-EV soluble fraction. The pass-through was applied to an Amicon Ultra-4 Centrifugal Filter Device MW.10k (Merck) to concentrate the non-EV soluble fraction. Total Exosome Isolation Reagent (ThermoFisher) was applied to the pre-EV fraction and incubated overnight at 4 °C. The precipitated EVs were collected by centrifugation at 10,000× *g* for 60 min at 4 °C. For biological assays, the EV fractions were eluted in 100 μL PBS (-). For protein assay, 10 × RIPA buffer containing 10% NP-40, 1% SDS, 5% deoxycholate in PBS (-), and a protease inhibitor cocktail (Sigma-Aldrich, St. Louis, MO, USA) was added to the EV fraction, incubated on ice for 15 min. The EV-derived protein samples were quantified with a principle of bicinchoninic acid (BCA) method using Micro BCA protein assay system (ThermoFisher). EV protein concentrations per cell were calculated at the time points of harvest. 

### 2.4. Mass Spectrometry

EV fraction was incubated in the presence of 1% SDS and 2.5 mM Tris (2-carboxyethyl)phosphine hydrochloride (ThermoFisher) for 10 min at 85 °C followed by alkylation with 12.5 mM iodoacetamide (Sigma-Aldrich) for 15 min at room temperature. Proteins were precipitated with acetone for 2 h at −30 °C and the resulting pellet was dispersed in 100 mM ammonium bicarbonate by ultrasonic treatment (three times for 30 s with intervals of 30 s) with a Bioruptor (Diagenode, Liège, Belgium). The protein suspension was subjected to digestion with trypsin (1 µg; Wako) for 14 h at 37 °C. Resulting peptides were analyzed by a QExactive mass spectrometer that was coupled with nano-LC (AdvanceLC; Michrom BioResources, Auburn, CA, USA) via a nano-electrospray source with a column oven set at 37 °C (AMR Inc., Gifu, Japan). Samples were injected to pre-column [L-column micro: 0.3 mm inner diameter, 5 mm length; Chemicals Evaluation and Research Institute (CERI), Japan] and separated by in-house made 20 cm column (inner diameter 100 µm, 3 µL-column; CERI, Japan) with a linear gradient (5%–30% B for 110 min, 30%–90% B for 1 min, and 90% B for 10 min, A: 0.1% formic acid, 2% acetonitrile, B: 0.1% formic acid, 99.9% acetonitrile) at a flow rate of 250 nL/min. The QExactive was operated in data-dependent acquisition mode. Scan ranges were set at *m*/*z* 375−1600 for MS spectra and *m*/*z* 200−2000 for MS/MS spectra, respectively. MS spectra were acquired at a resolution of 70,000 at *m*/*z* 400 after accumulation to a target value of 1 × 10^6^ with the maximum ion injection times for 60 msec. Up to the top 10, most abundant ions with charge 2+ or 3+ from the survey scan were selected with an isolation window of 1.5 h and fragmented by high energy collisional dissociation (HCD) with normalized collision energies of 25. MS/MS spectra were acquired at a resolution of 17,500 at *m*/*z* 400 after accumulation to a target value of 5 × 10^4^ with the maximum ion injection times for 120 msec. The acquired MS/MS spectra were analyzed by Proteome Discoverer 1.4 with the Mascot algorithm ver. 2.6.0 using an IPI human database (ver. 3.8.7). The search was performed with the following parameters: trypsin was selected as an enzyme used, the number of missed cleavages allowed was set as 3, and carbamidomethylation on Cys was selected as a fixed modification. Oxidized methionine was searched as variable modifications. Precursor mass tolerances were 10 ppm and tolerance of MS/MS ions was 0.02 Da. 

### 2.5. Electron Microscopy

TEM was carried out as described previously [3,20,48]. A 400-mesh copper grid coated with formvar/carbon films was hydrophilically treated. The EV suspension (5–10 μL) was placed on parafilm, and the grid was floated on the EV liquid and left for 15 min. The sample was negatively stained with 2% uranyl acetate solution for 2 min. EVs including exosomes on the grid were visualized with 5000–20,000 times magnification with an H-7650 transmission electron microscope (Hitachi, Tokyo, Japan) at Central Research Laboratory, Okayama University Medical School. To determine the size of EVs, TEM images were analyzed using Image J software. The diameters of 50 EVs were measured by taking the length of the widest point of each EV. Objects were limited to those greater than 50 nm.

### 2.6. EV Counting and Diameter Distribution Analysis

For EV diameter distribution analysis, 40 μL of EV fraction solved in PBS (-) was analyzed in a range between 0 and 10,000 nano-diameters in Zetasizer nano ZSP (Malvern Panalytical, UK) as described previously [3,20]. Alternatively, the diameter distribution of EVs was analyzed using ELS-8000 (Otsuka Electronics, Hirakawa, Japan) for a range of 1–10,000 nm with a setting of sidecut L20 as described previously [48].

For EV counting and diameter distribution analysis, the EV fractions were diluted 10-fold, passed through 0.22 μm-pore filters, and then analyzed using qNano particle analyzer (Meiwa Fosis, Osaka, Japan).

### 2.7. Cytotoxic LDH Assay

Cytotoxicity was measured using the index of lactate dehydrogenase (LDH) release from the cells and was expressed as a percentage of total cellular LDH activity. LDH activity was measured using an LDH cytotoxicity assay kit according to the manufacturer’s instructions (Nacalai Tesque, Kyoto, Japan). Cells were seeded at 5000 cells/well in 96-well plate and pre-cultured in RPMI1640 medium containing 10% FBS for 1 or 2 days. After the above-mentioned HSS for 0.5, 1.5, or 3 h, culture media were collected for the first LDH assay. Cells were washed with PBS (-), cultured in serum-free media for 24 h, and then conditioned media were collected. The collected culture media were transferred to another 96-well plate and incubated with substrate solution at RT for 20 min. The stop solution was added and absorbance (490 nm) was measured.

### 2.8. Cellular Morphology

Phase contrast cell photomicrographs were taken using Olympus CK30 equipped with a Digital Microscope Eyepiece Model MA88 (Premiere^®^) as described previously [22]. The rates of occurrence of cells with projections and round-shaped cells in cellular photomicrographs were counted by two researchers as blind experiments.

### 2.9. Promoter Analysis and Heat Shock Element

Promoter analysis was performed as described previously [60,75,76]. Briefly, DNA sequences of 5’-flanking regions and gene bodies (−5000 to +1000) of *Cdc37* in human (*Homo sapiens*), mouse (*Mus musculus*), rat (*Rattus norvegicus*), and fission yeast (*S. pombe*) were obtained from the Eukaryotic Promoter Database (EPD) [77]. Heat shock elements (HSE) based on the consensus sequence 5’-GAAxxTTCxxGAA-3’ and the reverse sequence 5’-TTCxxGAAxxTTC-3’ were searched using EPD.

### 2.10. Plasmid Constructs and Luciferase Assay

The *CDC37* promoter-luciferase reporter constructs (500+UTR and 200+UTR) [60], the overexpression constructs of heat shock factor 1 (HSF1) and dominant-negative (DN)-HSF1 [74], plasmid DNA co-transfection, and luciferase assay were described previously [60,74,78]. Briefly, cells were cultured in 96-well plates and a plasmid (25 ng reporter, 100 ng effector) was transfected with 0.4 µL FuGENE HD (Roche, Basel, Switzerland) per well at a cell confluence level of 50%–70%. The medium was changed at 16–20 h after transfection. At 40–48 h after transfection, 70 µL of the medium was aspirated, then 30 µL of Bright-Glo reagent (Promega, Madison, WI, USA) was added and mixed by pipetting. Cells were incubated for 5 min at 37 °C. The lysate (40 µL) was transferred to a 96-well white plate for measurement of luminescence.

### 2.11. RNA Interference

We designed siRNA sequences targeting mRNA of *CDC37, HSP90AA1*, or *HSP90AB1* (Table 1). For targeting each mRNA, a mixture of two types of siRNA duplex was used. The control non-targeting siRNA was purchased from Nippon Gene. Cells were transfected with siRNA using Lipofectamine RNAi MAX (Thermo Fisher). For single or double knockdown, cells were cultured in a 6-cm dish and 27 pmol siRNA was transfected with 8 µL of Lipofectamine RNAi MAX per dish for 24 h. The medium was replaced with serum-free fresh medium and the EV and whole cell lysate (WCL) were prepared at 48 h post-transfection. 

For triple knockdown, electroporation-mediated transfection was optimized and performed as described previously [3,60]. To optimize electroporation for each cell type, cells (1 × 10^5^ to 1 × 10^6^ cells), siRNA (40 pmol total), and serum-free medium were mixed to 100 µL total in a green cuvette with a 1-mm gap (NepaGene, Ichikawa, Tokyo, Japan) and set to NEPA21 Super Electroporator (NepaGene). Poring pulse was optimized between 100 V and 300 V for 2.5 or 5.0 msec pulse length twice with a 50 msec interval between the pulses and 10% decay rate with + polarity, as shown in a Supplementary Materials. The transfer pulse condition was five pulses at 20 V for 50 msec pulse length with 50 msec interval between the pulses and 40% decay rate with +/− polarity. After electroporation, cells were recovered in serum-containing media. PC-3 (5 × 10^5^ cells) was transfected with 40 pmol siRNA with poring pulse at 175 V for 2.5 msec pulse length twice and then cultured for 4 days for Western blotting. Alternatively, transfected PC-3 was cultured for 5 days and 1 × 10^6^ cells were subcutaneously injected to each SCID mouse as described below.

### 2.12. Whole Cell Lysate

Cells were lysed as described previously [79,80]. Briefly, cells were cultured until being sub-confluent and then washed with PBS (-), treated with 150–200 μL/dish of a 1× RIPA buffer containing 1% NP-40, 0.1% SDS, and 0.5% deoxycholate, and a protease inhibitor cocktail (Sigma) in PBS (-), and collected by using a cell scraper. Cells were further lysed by a 25G needle-syringe for 10 strokes and then incubated for 30 min on ice. For protein extraction from RWPE1 cells, cell lysis buffer (150 mM NaCl, 1% NP-40, 1% Na-deoxycholate, 0.1% SDS, 50 mM Tris, pH 7.5, 1 mM PMSF, 25 mM NaF, 2% Triton X-100) containing protease inhibitor cocktail was used. The lysate was centrifuged at 12,000× *g* for 20 min at 4 °C and the supernatant was used as a WCL. The WCL was diluted 10-fold and protein concentration was measured by using the Micro BCA protein assay system (ThermoFisher).

### 2.13. Western Blotting

Western blotting was performed as described previously [20,60]. In the HSS studies, protein samples corresponding to the same cell numbers were applied to each lane. The protein amount and cell numbers used in each experiment are described in Supplementary Materials. Briefly, the protein amount equivalent to 1 × 10^5^, 5 × 10^5^, 1 × 10^6^, or 3 × 10^6^ cells were loaded for analysis of EV fractions. The protein amount equivalent to 2 × 10^4^ or 6 × 10^4^ cells were loaded for analysis of WCL. The protein amount equivalent to 3 × 10^5^ cells were used for protein analysis in the non-EV cell culture supernatant. Protein samples were reduced with β-mercaptoethanol except for CD9 Western blotting. The samples were separated by SDS-PAGE in 4%–20% TGX-GEL (BioRad, Hercules, CA, USA) or 10% polyacrylamide gel and transferred to PVDF membrane using a semi-dry method. Anti-CDC37 (1:1000; Cell Signaling Technology (CST), Danvers, MA), anti-HSP90α (1:1000; GeneTex, Irvine, CA, USA), anti-HSP90β (1:1000; GeneTex), anti-CD9 (1:10,000 or 1:1000; MBL, Nagoya, Japan), anti-actin (1:200; Sigma), and HRP-conjugated anti-glyceraldehyde 3-phosphate dehydrogenase (GAPDH) (1:5000; FujiFilm Wako, Osaka, Japan) antibodies were used. The blots were visualized with ECL Plus Western blotting substrate (Pierce) or Immobilon Forte Western HRP substrate (Millipore, Burlington, MA, USA).

For studies of knockdown, EV addition, and EMT, equal amounts of protein samples in each Western blotting analysis (each 3 µg of protein samples for analysis of EV-CD9 and EV-GAPDH, and each 15 µg of protein samples for analysis of cellular proteins). For double knockdown studies, each 10 µg of protein samples for analysis of HSP90α, HSP90β, CDC37, E-cadherin, vimentin, and GAPDH was loaded. Protein samples were reduced with β-mercaptoethanol except for CD9 Western blotting, separated by SDS-PAGE in 4%–20% TGX-GEL (BioRad) or 10% polyacrylamide gel, and transferred to PVDF membrane using a semi-dry method. For EV addition studies, each 10 µg of protein samples for analysis of E-cadherin, N-cadherin, and GAPDH was loaded. Protein samples were reduced with β-mercaptoethanol, separated by SDS-PAGE in 8% polyacrylamide gel, and transferred to PVDF membrane using a wet method. In addition to the above-mentioned antibodies, anti-E-cadherin (1:1000, CST), N-cadherin (1:1000, CST), and anti-Vimentin (1:1000, CST) antibodies were used. The blots were visualized with ECL Plus Western blotting substrate (Pierce, ThermoFisher) or Immobilon Forte western HRP substrate (Millipore).

The images were quantified with the internal control of the actin or GAPDH protein levels and relative quantitative analysis was carried out based on the image band density ratio using ImageJ software (NIH, Bethesda, MD, USA). The quantitative values were shown in Supplementary Materials.

### 2.14. Tumor Xenograft

All animals were held under specific pathogen-free conditions. For chaperone triple-depletion, PC-3 cells (5 × 10^5^) were transfected with siRNA targeting mRNA of CDC37, HSP90α and HSP90β (17 pmol each) via electroporation and cultured for 5 days. PC-3 transfected with siRNA (1 × 10^6^ cells) was subcutaneously injected to each back of male SCID mice at 6- to 7-weeks old. The major axis (a) and minor axis (b) of tumors were measured with a caliper once a week until the day 99 post-injection period. The tumors were deemed to be ellipsoid and the volumes were calculated with a formula as follows: a tumor volume (V) ≒ 4πab^2^/3. These studies were carried out in strict accordance with the recommendations in the Guide for the Care and Use of Laboratory Animals of the Japanese Pharmacological Society. The protocol was approved by the Committee on the Ethics of Animal Experiments of the Okayama University (Permit Number: OKU-2016219).

### 2.15. Statistics

Data were expressed as the means ± SD unless otherwise specified. Statistical significance was calculated using GraphPad Prism (GraphPad, La Jolla, CA, USA). Comparisons of 2 were done with an unpaired Student’s *t*-test. Three or more mean values were compared using ANOVA Tukey’s multiple comparisons test.

## 3. Results

### 3.1. CRPC-Derived EVs Induce EMT in Normal Epithelial Cells

**Figure 1:**
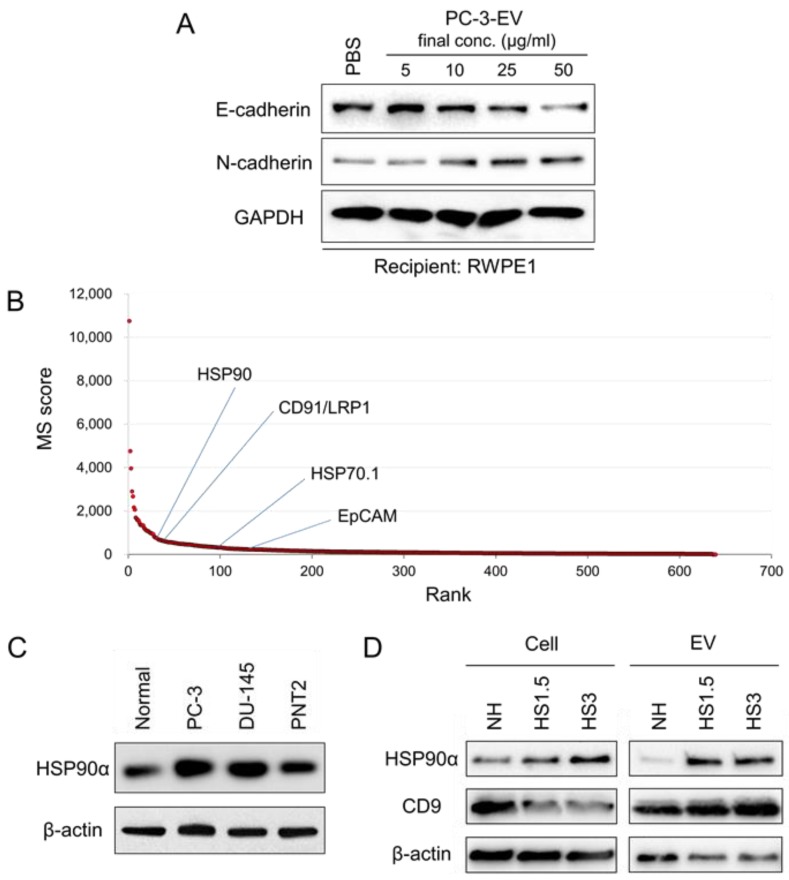
The pro-epithelial-to-mesenchymal transition (EMT) effect, proteome, and stress response of extracellular vesicles (EVs) released by PC-3 cells. (**A**) Western blot showing E-cadherin/CDH1, N-cadherin/CDH2, and GAPDH. EVs were prepared using 200-nm pore filter devices and polymer-based precipitation (PBP) method from culture media of PC-3. RWPE-1 cells were treated with the PC-3-derived EV at 5, 10, 25, or 50 µg/mL or PBS for 3 days and then lysed. (**B**) Scores of proteins identified by LC-MS/MS. (**C**) Western blot showing heat shock protein (HSP)90α and β-actin expressed in normal prostate epithelial cells, PC-3, DU-145, and PNT2 cells. The same set of protein samples was used previously [60]. (**D**) Western blot showing HSP90α, CD9, and β-actin in EVs and cells. PC-3 cells were stimulated with heat shock stress (HSS) for 1.5 or 3 h or non-heated (NH) and then cultured in serum-free media for 24 h to collect EVs and cell lysates.

We have shown that oral cancer-derived EVs induced EMT in normal epithelial cells [48]. We then hypothesized that cancer-derived EVs induced EMT in normal epithelial cells. We therefore aimed to establish whether PC-3-derived EVs could alter EMT properties in the normal prostate epithelial cell line RWPE1. E-cadherin, an established epithelial marker, was decreased by the addition of PC-3-derived EVs in a concentration-dependent manner (5, 10, 25 or 50 µg/mL) as compared to PBS-treated control (Figure 1A, Figure S1). In contrast, N-cadherin, an established mesenchymal marker, was increased by the addition of PC-3-derived EVs in a concentration-dependent manner. These data therefore suggested that PC-3-derived EVs could initiate EMT in the prostate epithelial cells.

### 3.2. The Proteome of sEV Released by PC-3 Cells

To establish a protein signature of sEV released by the CRPC cells, we performed a proteomic analysis of extracellular medium using LC-MS/MS. More than 700 protein species were thus identified, including molecular chaperones (total score: 795.5) such as HSP90α and HSP90β, extracellular signaling proteins (total score: 10759.0) such as thrombospondin 1, extracellular matrix (ECM) proteins (total score: 3964.7) such as fibronectin, agrin, and tenascin, cytoskeletal proteins (total score: 2070.1) such as actin, myosin, and keratin, ECM metabolic enzymes (total score: 1367.8), lipid and cholesterol metabolic proteins (1574.6), coagulation factors, proteinases, proteinase inhibitors, transmembrane proteins such as CD91/LRP1, metabolic and redox regulators, vesicle- and membrane-associated proteins such as annexin A2 and clathrin heavy chain (HC) (Figure 1B, Table 2).

We examined expression levels of HSP90α among normal prostate epithelial cells, PC-3, DU-145, and PNT2 cells. HSP90α was expressed at higher levels in PC-3 and DU-145 cells compared to normal prostate epithelial cells (Figure 1C). Next, we examined whether HSP90α could be altered in cells and EVs or released in soluble form upon proteotoxic damage exerted by HSS at 43 °C. HSP90α was abundantly released with EVs as well as non-EV extracellular HSP90 upon HSS for 1.5 or 3 h (plus 24 h recovery), while intracellular HSP90α was also increased (Figure 1D, Figure S2). As a control, we examined CD9 in the HSS study. Vesicular CD9 was increased upon the HSS, while intracellular levels decreased in the CRPC cells, suggesting that cellular CD9 was transmitted to EVs upon HSS. These findings indicate that HSP90α was released with EVs in response to proteotoxic stress, while non-vesicular HSP90α was also released in response to the HSS.

### 3.3. Stress Triggered Vesicle Release (200–500 nm) and Cell Morphological Changes

**Figure 2:**
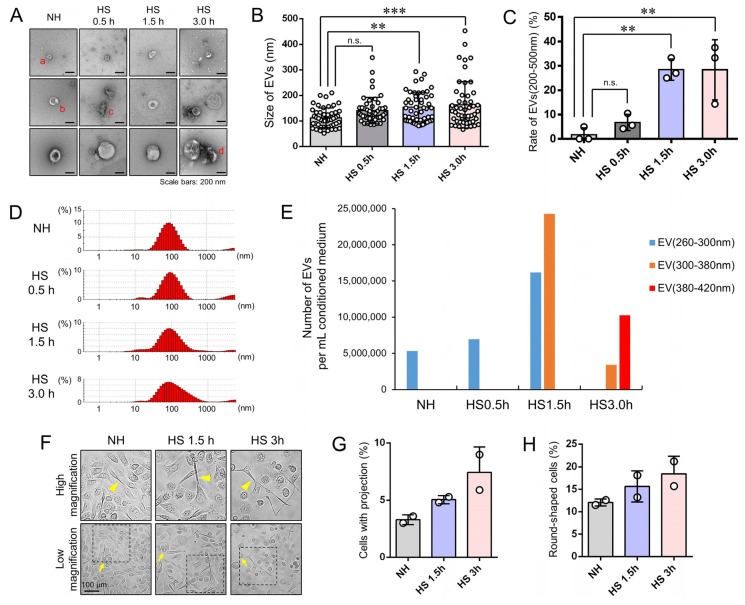
Release of EVs and membrane damage upon HSS. PC-3 cells were stimulated with HSS for 0.5, 1.5, or 3 h or NH, cultured for 24 h in serum-free media, from which EVs were then collected, and images were taken. (**A**) TEM images. (**a**), Cup-like EVs with clear walls of a potential lipid bilayer. (**b**), The wall of an EV was partially thick, which may be associated with another particle. (**c**), Membrane deformation was also seen in some EVs. (**d**), Crescent shapes are reminiscent of membrane brake of EVs. Scale bar, 100 nm. (**B**) Column scatterplot analysis of particle size. *n* = 50, mean ± SD. ANOVA and Tukey’s multiple comparison test was performed for statistics. **p < 0.01, ***p < 0.001 (vs. NH); n.s., not significant. (**C**) Column scatterplot analysis of the rate of EV(200–500 nm) upon HSS. *n* = 3 (random three fields), **p < 0.01; n.s., not significant. (**D**) Particle diameter distribution analysis using Zetasizer. (**E**) Numbers of EVs(260–300 nm), EVs(300–380 nm), and EVs(380–420 nm) per mL conditioned medium. qNano analyzer was used for counting EVs. (**F**–**H**) Cell morphological changes upon stress. (**F**) Representative images of cells. Top images were enlarged ones from the squares in the bottom images. Arrows, round-shaped cells. Arrowheads, cells with projections (or spindle shape). Scale, 100 µm. (**G**) Rate of cells with projections. (**H**) Rate of round-shaped cells.

Next, we determined whether the size and morphology of EVs released by the CRPC cells could be altered by HSS. Indeed, the size of EVs released from PC-3 cells without HSS was between 50 and 200 nm, while larger EVs sized between 200 and 500 nm increased in the medium upon HSS for 30 min, 1.5 h, and 3 h (Figure 2A–D; Figure S3 and Figure S4). The morphologies of the EVs corresponded basically to a cup-like shape, although the detailed shapes were various (Figure 2A). The prototypical EVs found under TEM were in a cup-like shape with clear walls potentially composed of a lipid bilayer, although the thickness of the EV walls varied (Figure 2Aa). The wall of an EV was partially thick, which may be associated with another particle (Figure 2Ab). Membrane deformation was also seen in some EVs (Figure 2Ac). Crescent shapes are reminiscent of membrane breakage of EVs (Figure 2Ad). These data suggested that the EVs of 50–200 nm were released from PC-3 cells without HSS, whereas the cells additionally released the larger EVs of 200–500 nm and membrane-damaged EVs upon stress, as a phenotype of the stressome.

Next, we examined whether numbers of EVs(260–300 nm), EVs(300–380 nm), and EVs(380–420 nm) were altered in the conditioned media by HSS. For this purpose, we used a qNano particle analyzer. The size of EVs tended to increase depending on the duration of HSS. The number of EVs(300–380 nm) was 16,177,300 vesicles per mL of the conditioned medium after 1.5 h of HSS (Figure 2E), whereas not detectable in NH and HSS(0.5 h) conditions. The number of EVs(380–420 nm) was 10,256,789 vesicles per mL of conditioned medium after 3 h of HSS, whereas not detectable in NH, HSS(0.5 h), or HSS(1.5 h) conditions. We also used another particle diameter analyzer ELS-8000 and confirmed that EVs(100–500 nm) were increased after HSS for 1.5 and 3 h (Figure S4). Thus, HSS increased larger EVs in culture supernatants of PC-3 cells as compared to the non-heated condition.

Along with the release of the stressome, the morphology of PC-3 cells was altered after HSS (Figure 2F). The rates of round-shaped cells and cells with projections (or spindle shape) were significantly increased after HSS (Figure 2F–H; Figure S5). 

These data suggested that cells underwent EMT with the loss of intercellular adhesion and junction under stress and stressome release was coupled with cell morphological changes.

### 3.4. EVs (200–500 nm) were Co-Released with LDH upon Membrane Damaging Stress 

**Figure 3:**
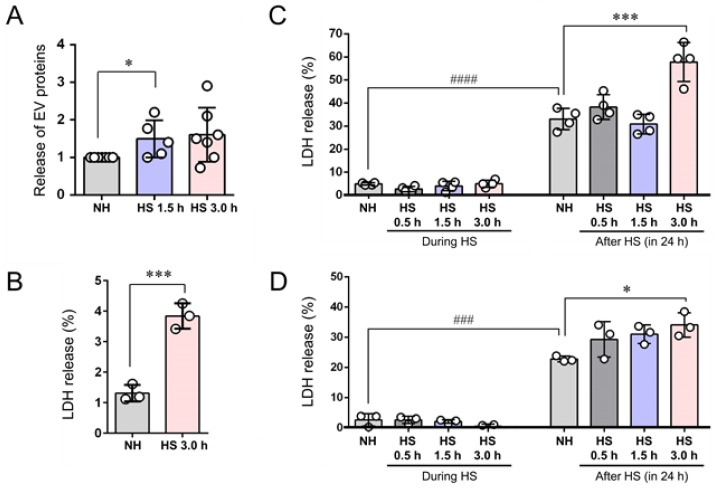
Release of EVs and membrane damage upon HSS. PC-3 cells were stimulated with HSS for 0.5, 1.5, or 3 h or NH, cultured for 24 h in serum-free media, from which EVs were then collected. (**A**) Release of EVs in response to stress. Protein concentrations of EV fractions were quantified using protein assay. The ratio compared to NH is plotted. *p < 0.05, *n* = 5 to 7. (**B**) Release of lactate dehydrogenase (LDH) upon HSS. PC-3 cells in the sparse condition were stimulated with or without HSS for 3 h and then LDH release was measured. (**C**,**D**) Column scatterplot analysis of LDH release. PC-3 cells were stimulated with HSS for 0.5, 1.5, or 3 h or NH, cultured for 24 h in serum-free media. The released LDH was measured. Data from two independent experiments are shown in C and D. Statistical analysis was carried out using ANOVA and Tukey’s multiple comparisons test. *n* = 3, ***p < 0.001, *p < 0.05, ^###^*p* < 0.001, ^####^*p* < 0.0001.

Membrane deformation and break of EVs were potentially induced by the HSS. The membrane deformation of EVs, as well as cells, could trigger the release of HSP90 from the cells and EVs. To establish the stress-responsive release of EVs, we next measured the protein concentration of EVs altered by the stress. EV release was significantly increased upon HSS in terms of protein concentration (Figure 3A). We next hypothesized that cell stress could damage cellular membrane permitting intracellular molecules such as HSPs and lactate dehydrogenase (LDH) to leak, while EVs such as ectosomes could be released by a physiological mechanism. To verify this, we next measured extracellular LDH released from the CRPC cells upon HSS. The release of LDH was increased by HSS for 3 h as compared to non-heated cells (Figure 3B–D). 

These results indicated that HSS induced the release of LDH from the CRPC cells potentially through membrane damage of the cells. sEV and HSP90 could be co-released with LDH upon the membrane damage caused by HSS.

### 3.5. Positive Regulation of CDC37 Promoter by HSF1

**Figure 4:**
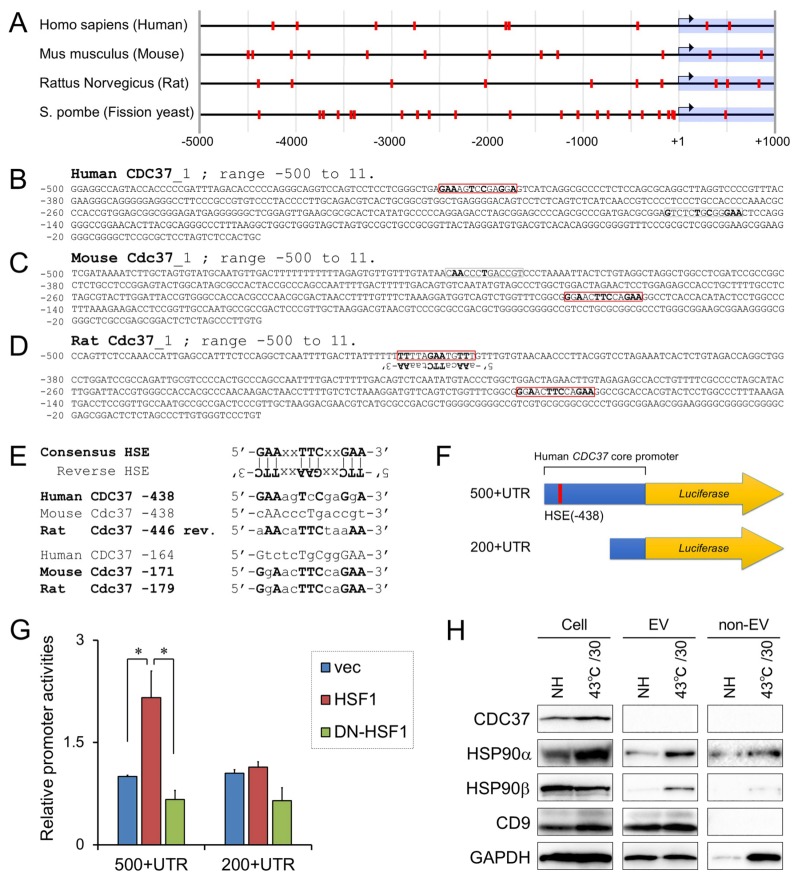
Heat shock response and roles of CDC37 in vesicular protein release. (**A**) Promoter analysis of *Cdc37* (−5000 to +1000) in human, mouse, rat, and fission yeast. Heat shock elements (HSE) were mapped as red boxes. (**B**–**D**) Mapping of HSE in *Cdc37* core promoter regions (−500 to transcription start site) in (**B**) human, (**C**) mouse, and (**D**) rat. HSEs were enclosed with red rectangles. (**E**) Alignment of HSE conserved among mammals. The HSE(−438) in human *CDC37* is conserved with a reverse HSE(−446) in rat *Cdc37*. Another HSE(−171) in mouse *Cdc37* is conserved with the HSE(−446) in rat *Cdc37*. (**F**) Schemes of human CDC37 promoter-luciferase reporter constructs. Promoter regions (500bp + UTR or 200bp + UTR) were connected with luciferase gene. UTR, 5′-untranslated region. (**G**) Activities of *CDC37* promoter regulated by heat shock factor 1 (HSF1). The *CDC37* promoter-reporter constructs were co-transfected with overexpression constructs of HSF1 or dominant-negative (DN)-HSF1 into DU-145 cells. *p < 0.05, *n* = 3. (**H**) Western blot showing CDC37, HSP90, CD9, and GAPDH altered by HSS in PC-3 cells, EVs, and non-EV fraction. PC-3 cells were stimulated with or without HSS (43 ℃ for 30 min), cultured in serum-free media for 24 h, and then cell lysate, EV, and non-EV fractions were collected.

CDC37 is a molecular chaperone that regulates the folding of kinases and nuclear receptors in association with HSP90 [61,62,81]. We therefore examined the potential role of this co-chaperone in the release of EVs. Many members of the HSP family are stress-responsive, i.e., characteristically inducible by heat shock under the control of heat shock-responsive transcription factor 1 (HSF1) [74,82], but also by hypoxia, and starvation stress. However, it has not been fully established whether CDC37 was stress-inducible or not. HSF1 binds to heat shock elements (HSE: consensus sequence, 5′-GAAxxTTCxxGAA-3′ and reverse consensus sequence 5′-TTCxxGAAxxTTC-3′) in promoter regions of target genes. We therefore searched HSE in *Cdc37* genes in eukaryotes. Several HSE were scattered around the transcription start sites (TSS) in 5′-flanking regions and gene bodies (−5000 to +1000) of *Cdc37* gene in human, mouse, and rat (Figure 4A), while HSE were more abundant in the same range of *Cdc37* gene in fission yeast. In the core promoter regions (−500 to +1) of the *Cdc37* gene in these mammals, two HSE at the −438 and −171 positions were conserved between species (Figure 4B–D). The HSE(−438) 5′-GAAagTcCgaGgA-3′ in human *CDC37* was conserved with a corresponding HSE(−446) 5′-aAAcaTTCtaaAA-3′ in rat *Cdc37*, while another HSE(−171) 5′-GgAacTTCcaGAA-3′ in mouse *Cdc37* was 100% conserved with the HSE(−179) 5′-GgAacTTCcaGAA-3′ in rat *Cdc37* (Figure 4E).

To examine whether the *CDC37* promoter could be regulated by HSF1, we performed the reporter assay using the *CDC37* promoter-luciferase constructs. We here used DU-145 prostate adenocarcinoma cells, in which endogenous CDC37 level was not fully induced as compared to the PC-3 cells [60]. The *CDC37* promoter (500+UTR) containing the HSE(−438) was activated by HSF1 overexpression in DU-145 cells, whereas dominant-negative HSF1 (DN-HSF1) failed to activate the *CDC37* promoter (Figure 4F,G). Moreover, another *CDC37* promoter construct (200+UTR) without HSE did not respond to HSF1 overexpression. These data indicated that HSF1 mediates cell stress signal to the *CDC37* gene.

### 3.6. Stress-Responsive Induction of CDC37, CD9, and Extracellular HSP90α and GAPDH

We next examined whether CDC37, HSP90, and CD9 are inducible by HSS and released from PC-3 cells. As expected, CDC37 was induced by HSS (43 °C, 30 min), but not found in either the extracellular or non-vesicular fractions (Figure 4H, top; Figure S6). HSP90α, a known inducible type of HSP, was increased by HSS intracellularly, in EVs and the non-vesicular extracellular fraction (Figure 4H, second row). HSP90β, the constitutively expressed HSP90 ortholog was increased in the EV fraction upon the HSS, while intracellular HSP90β was decreased, suggesting that HSP90β could be transmitted from the cells to EV fraction upon the HSS (Figure 4H, third row). CD9 was increased in the cells and the EV fraction upon the HSS (Figure 4H, fourth row). Extracellular GAPDH was increased coordinately with the increase of extracellular HSP90α (Figure 4H, bottom row).

These data indicated that the cell stress-induced CDC37, extracellular HSP90α and GAPDH, and CD9-positive EVs, coordinately.

### 3.7. CDC37, a Stress-Responsive Protein Essential for the Release of EVs

**Figure 5:**
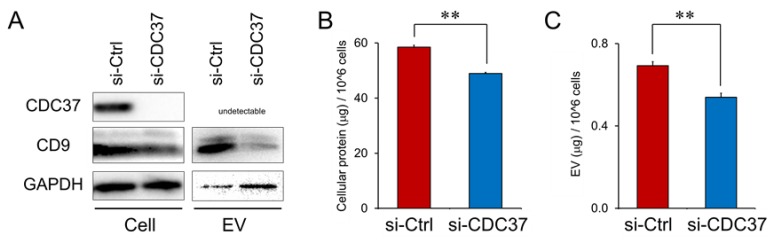
Inhibition of proteostasis and CD9 in EVs by the knockdown of CDC37. The siRNA targeting CDC37 or non-targeting control siRNA was transfected into PC-3 cells. Cell lysate and EVs were prepared at 48 h after the transfection. (**A**) Western blot showing CDC37, CD9, and GAPDH in PC-3 cells. EVs were prepared using 200-nm pore filter devices and PBP method. (**B**) Cellular protein concentration per million cells. **p < 0.01, *n* = 3. (**C**) EV protein concentration per million cells. **p < 0.01, *n* = 3.

These data prompted us to hypothesize that CDC37, although not secreted itself, could be involved in the secretion of CD9-positive vesicles. To verify this hypothesis, we next asked whether the CD9 levels in the cellular and EV fractions could be altered by CDC37 knockdown using siRNA. CDC37 knockdown markedly decreased the CD9 level in the cellular and EV fractions (Figure 5A and Figure S7). Coordinately, cellular protein concentrations per cell were significantly decreased by CDC37 knockdown as compared to the control siRNA condition (Figure 5B). EV protein concentration per cell was also significantly decreased by CDC37 knockdown (Figure 5C). 

These data suggested that CDC37 was essential for proteostasis, CD9 synthesis, and the release of CD9-containing vesicles from the CRPC cells.

### 3.8. CDC37 is Essential for EMT in the CRPC Cells

**Figure 6:**
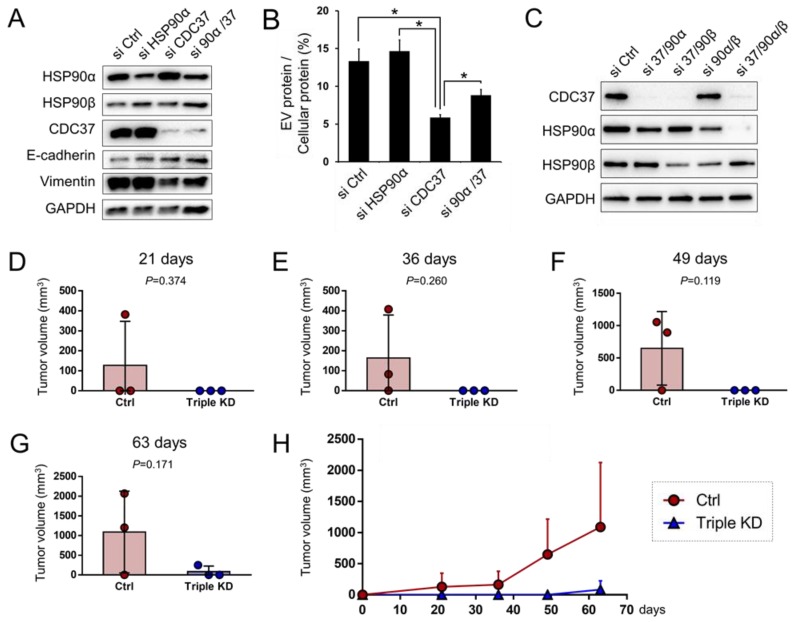
EMT properties, vesicle release, and tumorigenicity were declined by targeting CDC37 and HSP90. (**A**,**B**) PC-3 cells were transfected with siRNA targeting CDC37, HSP90α, their combination, or non-targeting control siRNA for 48 h and then cell lysates and EVs were collected. (**A**) Western blot showing CDC37, vimentin, E-cadherin, HSP90, and GAPDH. (**B**) The ratio of EV vs. cellular protein concentrations. *p < 0.05, *n* = 3. (**C**) Western blot showing CDC37 and HSP90 reduced by double or triple knockdown. siRNA targeting CDC37, HSP90α, and HSP90β or non-targeting control siRNA were transfected into PC-3 cells and then cells were lysed at 48 h post-transfection. Efficiencies of double or triple knockdown were tested. (**D**–**H**) In vivo tumorigenesis declined by triple chaperone knockdown. PC-3 cells were transfected with triple siRNA combination (CDC37, HSP90α, HSP90β) or non-targeting control siRNA and then cells were subcutaneously xenografted to SCID mice. Tumor volumes at (**D**) day 21, (**E**) day 36, (**F**) day 49, and (**G**) day 63 post-xenograft periods were analyzed by scatter-plotting. Data are expressed as mean ± SD, *n* = 3. (**H**) The time-course graph of tumor volumes altered between triple chaperone knockdown vs. the control siRNA groups. Data are expressed as mean + SD, *n* = 3.

HSP90α is an inducible type of HSP in stressed conditions and cancer, while HSP90β is a constitutively expressed type of HSP [14,27]. We next examined whether both CDC37 and HSP90α could be involved in EMT properties in PC-3 cells. We here investigated the vimentin level, a well-established mesenchymal marker. The knockdown of CDC37 markedly decreased vimentin and increased E-cadherin levels in the CRPC cells (Figure 6A and Figure S8), indicating that CDC37 is an essential factor in EMT. The knockdown of HSP90α increased E-cadherin, suggesting that HSP90α is also crucial in EMT. The knockdown of HSP90α also increased HSP90β, a potentially compensatory response in the loss of chaperone.

Next, we examined whether CDC37 and HSP90α could be involved in the release of EVs from PC-3 cells. The knockdown of CDC37 significantly decreased the fraction of EV protein compared with cellular protein concentration, suggesting that EV release was inhibited by CDC37 knockdown (Figure 6B). EVs(200–1000 nm) were reduced by the knockdown of CDC37/HSP90α (Figure S9) along with the loss of EV protein concentration.

These data indicated that CDC37 was essential for proteostasis, EV protein release, and EMT in prostate cancer cells.

### 3.9. Triple Targeting of CDC37, HSP90α, and HSP90β Declines the Tumorigenicity of CRPC In Vivo

We have experienced that knockdown of a chaperone could often trigger the compensatory induction of another chaperone. We therefore next examined double knockdown and triple knockdown of the chaperone trio- CDC37, HSP90α, and HSP90β. Each combination of siRNA successfully reduced their respective targets, while triple siRNA combination markedly reduced the CDC37, HSP90α, and HSP90β trio (Figure 6C and Figure S10). Previous studies have shown that PC-3, a CRPC cell line, was the most tumorigenic prostate cancer cell line as compared to the other established prostate carcinoma cell lines [4]. We next examined whether the triple knockdown of the chaperone trio (CDC37, HSP90α, HSP90β) altered in vivo tumorigenesis of the CRPC cells. The triple knockdown of the chaperone trio tended to inhibit in vivo tumorigenesis of PC-3 cells (Figure 6D–H). Tumor incidence was 2/3 in the control group, while 0/3 in the triple knockdown group until day 56, suggesting the triple knockdown of the chaperones was anti-tumorigenic.

These results indicated that members of the chaperone trio (CDC37, HSP90α, HSP90β) were essential for tumorigenicity of the CRPC cells.

## 4. Discussion

Cell extrinsic molecules secreted by both tumor and infiltrating normal cells may play key roles in conditioning the tumor environment and enhancing malignancy. For instance, infiltrating macrophages and fibroblasts may supply key growth factors and ECM molecules enhancing growth and metastasis [83,84,85]. Tumors may also condition their environments with molecules such as HSPs to supply essential chaperones to recipient cells and trigger receptor-mediated signaling [19,86]. However, the exact details of the tumor chaperone network are not yet defined. Our study demonstrated that HSP90 and CDC37 are essential for a key component of the network, stressome release which permits the exit of HSPs and promotes tumor progression in CRPC. The release of EVs and HSP90 from the CRPC cells was a major aspect of the resistance-associated secretory phenotype (RASP). Moreover, we show intracellular CDC37 to be essential for EMT-coupled EV release from CRPC cells, which may constitute a key first step to understanding the mechanisms underlying HSP-loaded EV secretion. There are more than 10 co-chaperones of HSP90, which individually carry out distinct cooperative roles with HSP90 in cells [87]. CDC37 is a definitive kinome chaperone assisting in the folding of protein kinases such as SRC, many receptor tyrosine kinases (RTK), their downstream Ras/Raf/MEK/ERK signaling pathway, and PI3K-AKT signaling pathway, which promote EMT [88,89,90,91]. Among such key kinases, a recent study showed that SRC in endosomal membranes promoted exosome secretion and tumor progression [92]. Moreover, activation of the epidermal growth factor receptor (EGFR) promoted the secretion of EGFR-rich EVs, whose transmission to recipient cells promoted EMT and metastasis [48,93,94,95,96]. LRP1/CD91 could be another RTK, a receptor involving the kinase-specialized CDC37/HSP90 chaperone complex and activates the MEK-ERK signaling pathway [97]. CDC37 could positively regulate EMT and the release of EVs through permitting the function of these protein kinases. Consistently, recent studies have shown that EMT in cancer cells was often coupled with the release of EVs and drug resistance [22,98,99].

**Figure 7:**
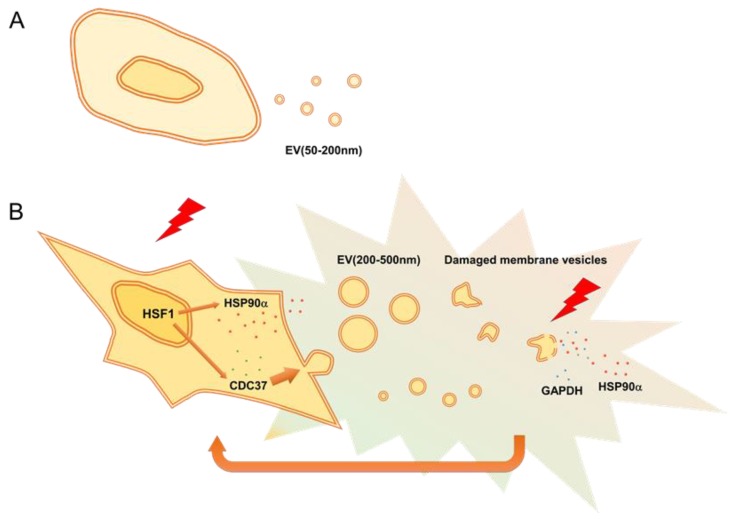
Graphical abstract. (**A**) Prostate cancer cells release EVs(50–200 nm) that contain HSP90α and transform normal epithelial cells by inducing EMT. (**B**) Cellular stress such as HSS activates HSF1, which induces the production of CDC37 and HSP90α via transcriptional activation (left). CDC37 is essential for proteostasis and the release of CD9-containing EVs. HSS for 1.5–3 h also triggers the production of larger EVs(200–500 nm) and membrane damage of EVs, from which HSP90α and GAPDH could be leaked. Therefore, we here define “stressome” as cell stress-induced all secretion products including EVs, membrane-damaged vesicles, and factors released from EVs and cells. Stressome may promote tumor progression.

Our studies also touch upon the induced release of HSP90 in the CRPC cells. Several studies, including ours, have indicated that HSP90 is essential in stress resistance in cancer cells [4,19,20,50,52]. Notably, extracellular HSP90 plays key roles in tumor progression and metastasis as well as immune surveillance [27]. It has been shown that HSP90α was an inducible type of HSP in a stressed condition and cancer, while HSP90β was a constitutively expressed type of HSP [14,27]. However, mechanisms by which HSP90α or HSP90β are released from vesicles or cells had not well-investigated before our study. We demonstrated HSP90α to be robustly inducible by HSS and efficiently released as a cargo of EV as well as in the form of EV-free HSP90. In contrast, the HSP90β ortholog was not markedly inducible but was transmitted to EVs upon HSS and barely dissolved in the extracellular fluid. Notably, membrane-damaged EVs, EVs(200–500 nm), HSP90α, and GAPDH were co-released upon HSS, suggesting that vesicular membranes were damaged by the stress, which allowed the release of cargos, including HSP90α and GAPDH, from the damaged EVs (Figure 7, graphical abstract). Therefore, we here define “stressome” as cell stress-induced all secretion products released from cells and EVs. Stressome markers include EVs(200–500 nm), damaged membrane remnants, extracellular HSPs, and extracellular GAPDH. Indeed, extracellular HSPs have been known to exhibit the functions of DAMPs. Moreover, membrane-bound HSPs can be associated with stress-responsive EVs [27]. Otherwise, the production of ectosomes (100–500 nm) requires budding and shedding of the cell membrane, which can create membrane damage on vesicles and cells. Moreover, it has been shown that cell motility and stem cell properties induced by the EMT required destabilization of lipid rafts [100]. Therefore, it is conceivable that heat shock triggers the co-release of ectosomes, HSP90, and LDH through membrane damage of vesicles and cells and destabilization of lipid rafts. A recent study showed that HSP90 mediated membrane deformation and exosome release [101]. In our study, knockdown of CDC37 significantly decreased the release of EV proteins, including CD9, from the CRPC cells. Therefore, it was conceivable that the chaperone activity of CDC37 was essential for proteostasis and the release of sEV proteins. CD9 has been known as a marker of exosomes, small vesicles secreted from cells via exocytosis. However, recent studies pointed out the heterogeneity of EVs and exosomes [29,102,103,104,105]. Therefore, for example, some EVs contained many CD9, while the other EVs might not contain CD9. Our study indicated that HSS increased CD9 in cells, involving the increase in CDC37. However, CD9 might not be incorporated in EVs or exosomes in only 30 min of HSS. Thus, CD9, a canonical marker of exosomes, showed different dynamics from HSP90α, a marker of stressome.

It is also clear that the properties of 3D tumors in vivo might be largely different in many aspects from 2D-cultured cells in vitro. Single targeting of CDC37 did not inhibit in vivo tumorigenicity, while triple targeting of CDC37/HSP90α/HSP90β markedly inhibited tumorigenicity of CRPC cells. Consistently, HSP90α was markedly released from 3D tumoroids of PC-3 cells, which resemble a miniaturized tumor in vitro. Thus, the chaperone trio composed of CDC37 and HSP90α/β was crucial for tumorigenicity of CRPC cells. Notably, the tumorigenicity of CRPC cells is a key property for recurrence and metastasis in this type of prostate cancer. In the androgen insensitivity, intracellular kinase signaling pathways can be of higher priority required for tumor progression. This logic is consistent with elevated expression of CDC37, a kinome chaperone, in CRPC as compared to prostate adenocarcinoma and with the anti-tumor effect of the triple chaperone depletion. 

Our data also indicated that HSF1, a mediator of the stress response, positively regulates the *CDC37* gene expression. We recently demonstrated that myeloid zinc finger 1 (MZF1) and SCAND1 reciprocally regulated *CDC37* gene expression in prostate cancer. In this study, a gain of MZF1, as well as a loss of SCAND1, were crucial for *CDC37* expression. In addition, intracellular matrix metalloproteinase (MMP) 3 as a transcription factor cooperated with HSF1-mediated stress response [74,78,106]. Thus, HSF1 can coordinately activate the *CDC37* gene along with other transcription factors, while especially playing a key role in stress response and resistance. Indeed, the overexpression of a DN-HSF1 construct inhibited aneuploidy in prostate carcinoma cells [107]. Thus, HSF1 activation of *CDC37* could be crucial for exosome release as well as EMT. 

## 5. Conclusions

We define “stressome” as a cell stress-induced secretory program including EVs(200–500 nm), membrane-damaged vesicles and remnants, and extracellular HSP90 and GAPDH. Our data also indicated that CDC37 is crucial for the release of vesicular proteins and tumor progression in prostate cancer.

## Figures and Tables

**Table 1:** List of sequences of siRNA.

Name of siRNA Oligonucleotide	Sequence (5’ to 3’)
hCDC37.NM7065-433 sense	gcaagaaggagaagagcauTT
hCDC37.NM7065-433 antisense	augcucuucuccuucuugcTT
hCDC37.NM7065-584 sense	gaaacagaucaagcacuuuTT
hCDC37.NM7065-584 antisense	aaagugcuugaucuguuucTT
hHSP90AA1.NM5348-415 sense	gcugcauauuaaccuuauaTT
hHSP90AA1.NM5348-415 antisense	uauaagguuaauaugcagcTT
hHSP90AA1.NM5348-2010 sense	caaacauggagagaaucauTT
hHSP90AA1.NM5348-2010 antisense	augauucucuccauguuugTT
hHSP90AB1-NM_001271971.1-1353 sense	cagaagacaaggagaauuaTT
hHSP90AB1-NM_001271971.1-1353 antisense	uaauucuccuugucuucugTT
hHSP90AB1-NM_001271971.1-1754 sense	gaagagagcaaggcaaaguTT
hHSP90AB1-NM_001271971.1-1754 antisense	acuuugccuugcucucuucTT

**Table 2:** The proteome of EVs released from PC-3 cells.

Class	Summed Scores	Top Hit Proteins	Number of Protein Types
Extracellular signaling	10759.0	TSP1, LGALS3BP	37
ECM proteins	3964.7	Fibronectin, Agrin, Tenacin	53
Cytoskeletal	2070.1	Actin, Myosin, Keratin	40
ECM metabolic enzymes	1367.8	PLOD1, PXDN	6
Lipid and cholesterol metabolism	1574.6	ApoB, ApoA-I	9
Coagulation	1173.8	F2	4
Chaperones, HSP	795.5	HSP90-alpha, Hsp90-beta, HSP70.1	23
Proteinases, proteinase inhibitor	721.6	tPA	17
Transmembrane	582.5	CD91/LRP1, Neuropilin 1	53
Metabolism and Redox	532.7	PKM	51
Vesicle- and membrane-associated	499.6	Annexin A2, Clathrin HC	16
Translational regulators	483.8	EF2	9
Isomerases	285.4	PPIA	7
Hormones	317.4	Inhibin beta	3
Lysosome	364.5	MAN2B1	2
Histone, transcriptional, epigenetic	237.2	H4, H1.4	11
Proteasome	221.0	PSMA7	19
Molecular Traffic	218.6	KIF23	6
G proteins	172.2	RACGAP1, Rac1	13
Calcium signaling	142.8	NBD1	6
Intracellular signaling	135.8	Catenin D1	4
Kinases	133.6	PGK1	7
Phosphatase	94.5	PP2A	6
Ribosome	86.1	Ribosomal protein S28	13
Antimicrobial peptide	47.8	Dermcidin	1
Miscellaneous	340.7	PSAPL1	54
Putatively serum-derived	2673.7	C3	14
Uncharacterized proteins	1544.6	u.c.p.	155

## Supplementary Materials

The following are available online at https://www.mdpi.com/2073-4409/9/3/755/s1, Figure S1. Western blotting showing E-cadherin, N-cadherin, and GAPDH, supporting Figure 1A; Figure S2. Western blotting showing EV-HSP90α, -CD9, -β-actin, Cell-HSP90α, -CD9, and –β-actin, supporting Figure 1D; Figure S3. Full views of TEM, supporting Figure 2A,B; Figure S4. Heat shock stress-induced EVs(100–500 nm) in culture media of PC-3 cells; Figure S5. Full views of cellular photomicrographs, supporting Figure 2F–H; Figure S6. Western blotting showing CDC37, HSP90α, HSP90β, CD9, and GAPDH, supporting Figure 4H; Figure S7. Western blotting showing EV-CD9, EV-GAPDH, Cell-CDC37, and Cell-GAPDH, supporting Figure 5A; Figure S8. Western blotting showing HSP90α, HSP90β, CDC37, E-cadherin, Vimentin, and GAPDH, supporting Figure 6A; Figure S9. Knockdown of CDC37 and HSP90alpha reduced EV(200–1000 nm); Figure S10. Western blotting showing CDC37, HSP90α, HSP90β, and GAPDH, supporting Figure 6C.

## Abbreviations

CDC37	cell division control 37
CRPC	castration-resistant prostate cancer
DAMP	damage-associated molecular pattern, danger-associated molecular pattern
ECM	extracellular matrix
EMT	epithelial-mesenchymal transition
EV	extracellular vesicle
GAPDH	glyceraldehyde-3-phosphate dehydrogenase
HSE	heat shock element
HSF1	heat shock factor 1
HSP	heat shock protein
HSS	heat shock stress
LDH	lactate dehydrogenase
LRP1	low-density lipoprotein-related protein 1
MS	mass spectrometry
NET	neuroendocrine tumors
RASP	resistance-associated secretory phenotype
sEV	small extracellular vesicle
siRNA	small interfering RNA
TEM	transmission electron microscopy
