# Supplementary material for: Cell Stress Induced Stressome Release Including Damaged Membrane Vesicles and Extracellular HSP90 by Prostate Cancer Cells
Source: Cells. 2020 Mar 19;9(3):755. doi: 10.3390/cells9030755 (PMC7140686; doi:10.3390/cells9030755)
Supplement: Supplementary file 1 [file cells-09-00755-s001.pdf]

Supplementary Materials: Cell Stress Induced Stressome Release Including Damaged Membrane Vesicles and Extracellular HSP90 by Prostate Cancer Cells

Takanori Eguchi, Chiharu Sogawa, Kisho Ono, Masaki Matsumoto, Manh Tien Tran, Yuka Okusha, Benjamin J. Lang, Kuniaki Okamoto and Stuart K. Calderwood

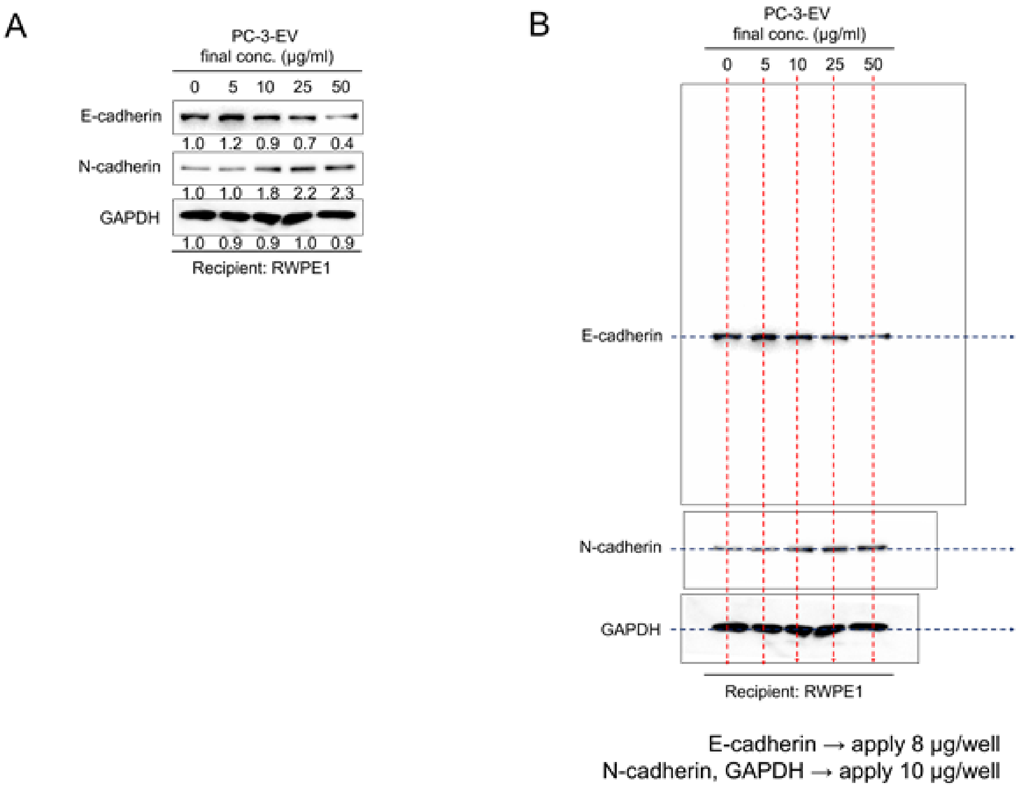

Figure S1. Western blotting showing E-cadherin, N-cadherin, and GAPDH, supporting Figure 1A. (A) Relative intensities of the bands. (B) Full images of the western blotting. For E-cadherin, 8 μg each protein samples were applied. For N-cadherin and GAPDH, 10 μg each protein samples were applied per lane.

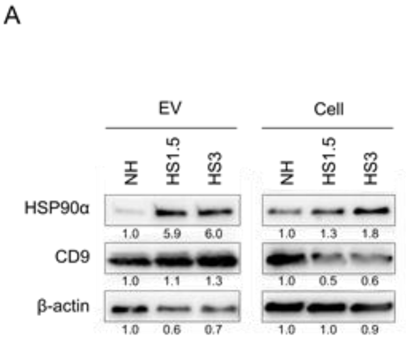

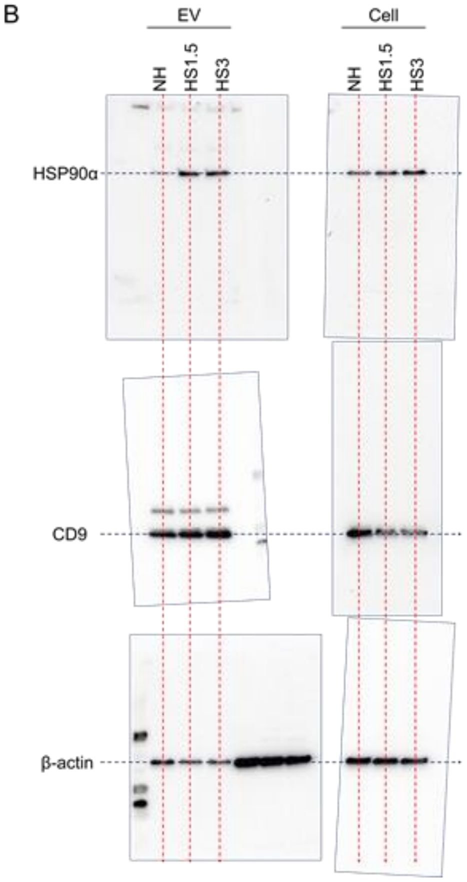

C

HSP90α						
	EV			Cell		
	NH	HS 1.5	HS 3	NH	HS 1.5	HS 3
Protein (μg)	4.14	4.50	6.54	10.58	4.06	8.16
Cells	3x10^6			2x10^4		

CD9						
	EV			Cell		
	NH	HS 1.5	HS 3	NH	HS 1.5	HS 3
Protein (μg)	1.38	1.50	2.18	31.7	12.2	24.5
Cells	1x10^6			6x10^4		

β-actin						
	EV			Cell		
	NH	HS 1.5	HS 3	NH	HS 1.5	HS 3
Protein (μg)	1.38	1.50	2.18	10.58	4.06	8.16
Cells	1x10^6			2x10^4		

Figure S2. Western blotting showing EV-HSP90α, -CD9, -β-actin, Cell-HSP90α, -CD9, and –β-actin, supporting Figure 1D. (A) Relative intensities of the bands. (B) Full images of the western blotting. (C) Amounts of proteins applied for each western blotting analysis were shown in the table. The protein samples corresponding to same cell numbers were applied each lane.

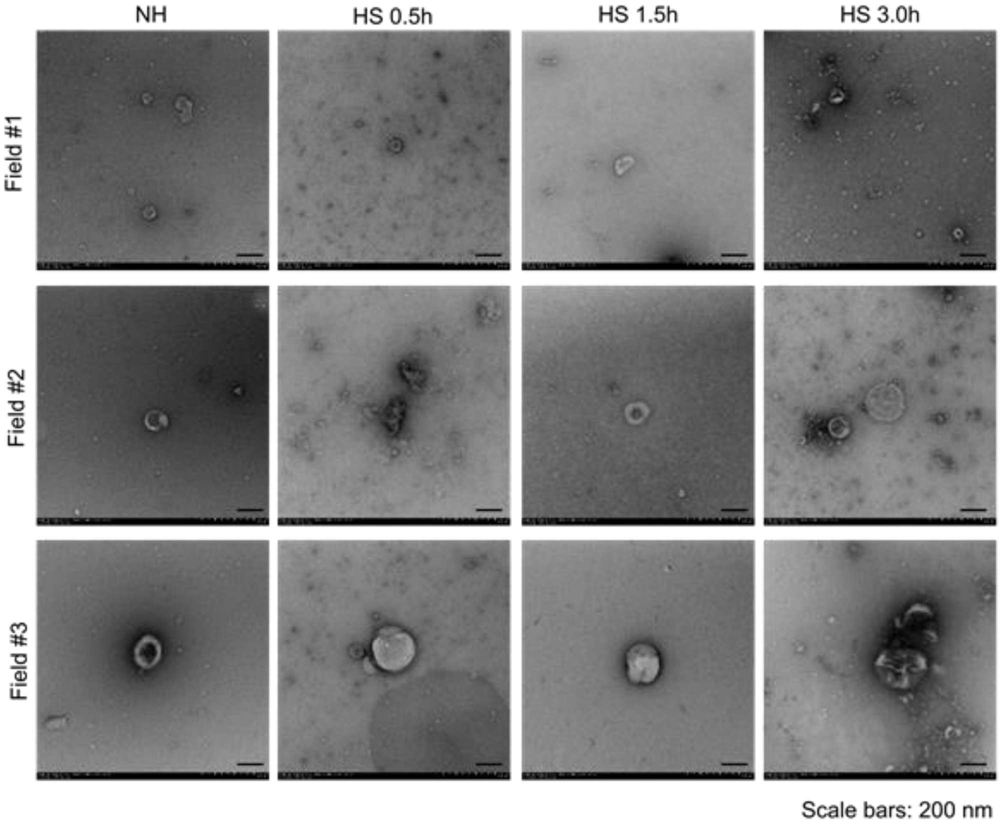

Figure S3. Full views of TEM, supporting Figure 2A, B. Scale bars, 200 nm.

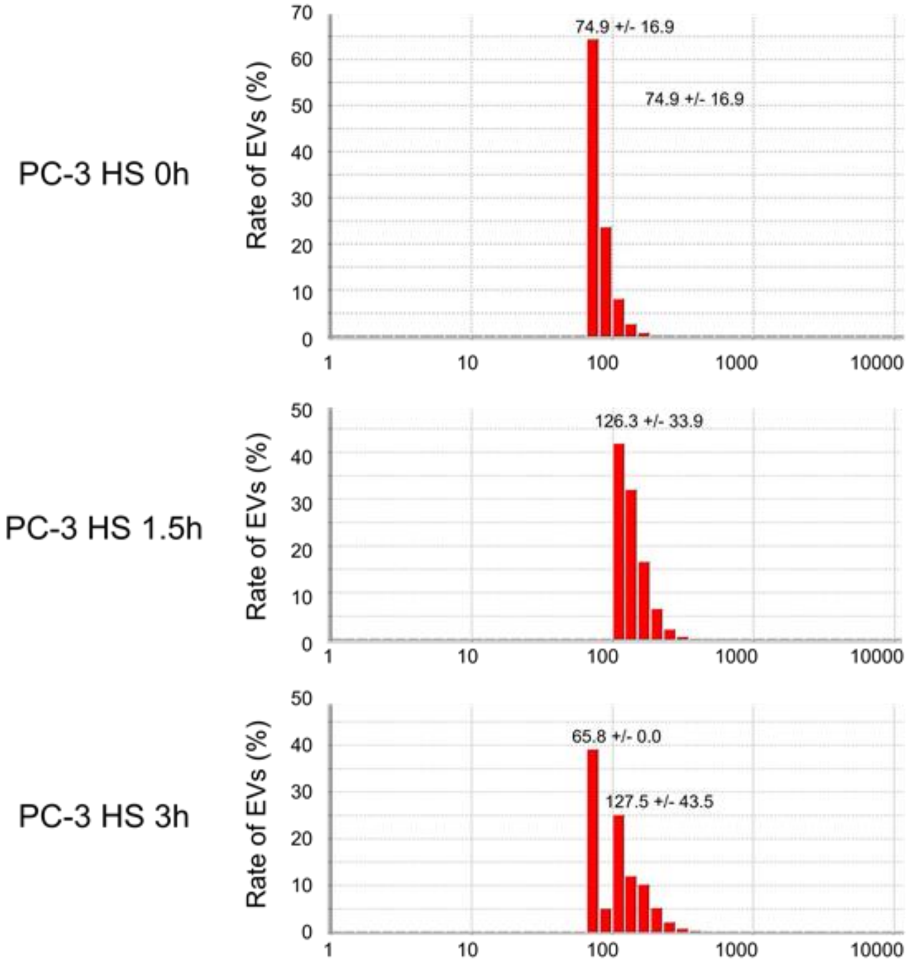

Figure S4. Heat shock stress induced EVs(100-500nm) in culture media of PC-3 cells. Cells were heat-shocked for 0, 1.5 or 3 hours and recovered for 24 hours in serum-free media, from which EVs were prepared. Diameters of EVs were analyzed using ELS-8000 (Otsuka Electronics, Hirakawa, Japan).

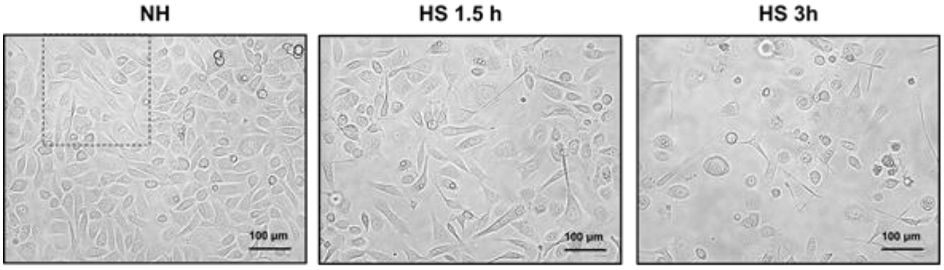

Figure S5. Full views of cellular photomicrographs, supporting Figure 2 F to H.

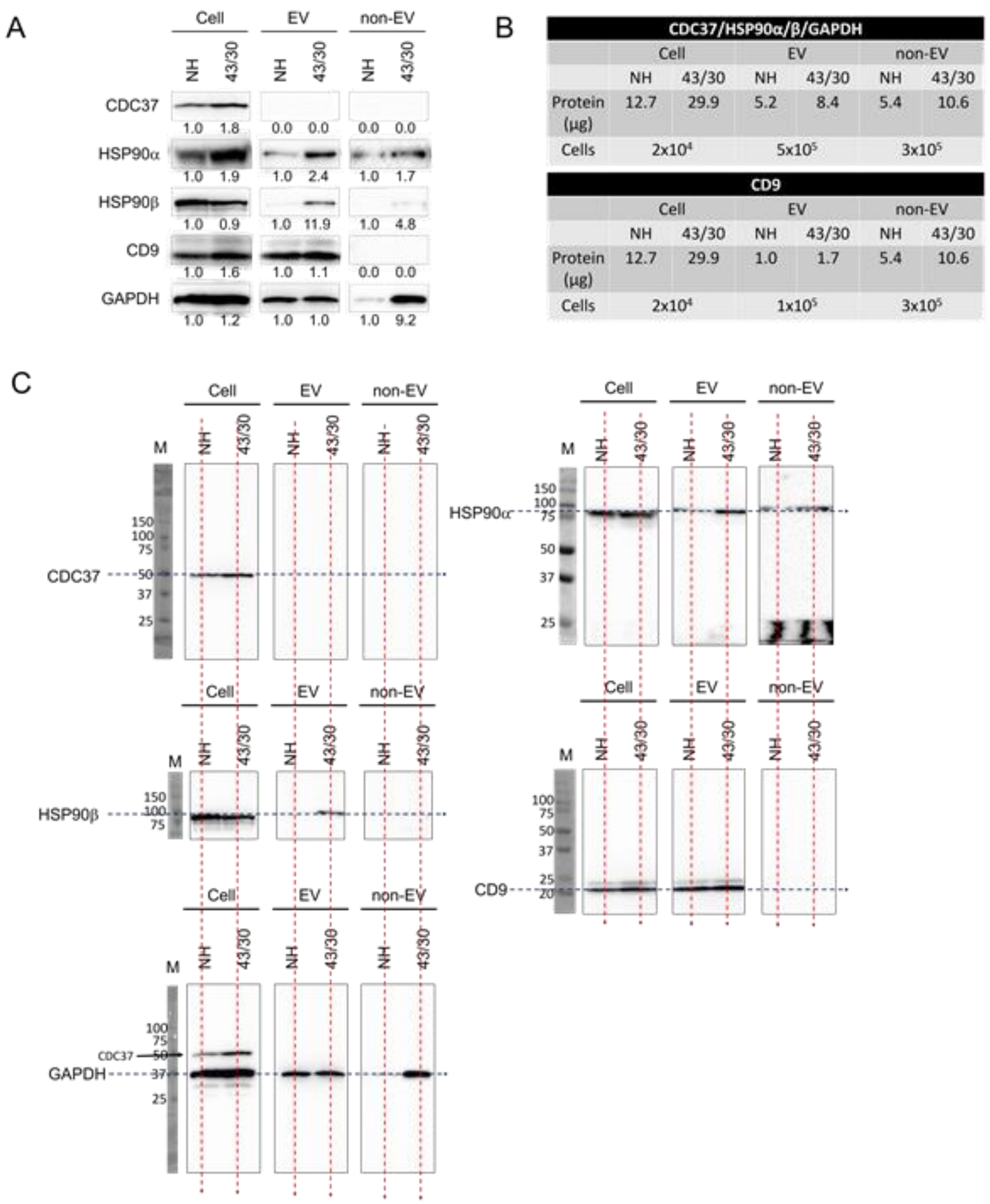

CDC37/HSP90α/β/GAPDH						
	Cell		EV		non-EV	
	NH	43/30	NH	43/30	NH	43/30
Protein (μg)	12.7	29.9	5.2	8.4	5.4	10.6
Cells	2x10⁴		5x10⁵		3x10⁵	

CD9						
	Cell		EV		non-EV	
	NH	43/30	NH	43/30	NH	43/30
Protein (μg)	12.7	29.9	1.0	1.7	5.4	10.6
Cells	2x10⁴		1x10⁵		3x10⁵	

Figure S6. Western blotting showing CDC37, HSP90α, HSP90β, CD9, and GAPDH, supporting Figure 4H. (A) Relative intensities of the bands. (B) Amounts of proteins applied for each western blotting analysis were shown in the table. The protein samples corresponding to same cell numbers were applied each lane. (C) Full images of the western blotting.

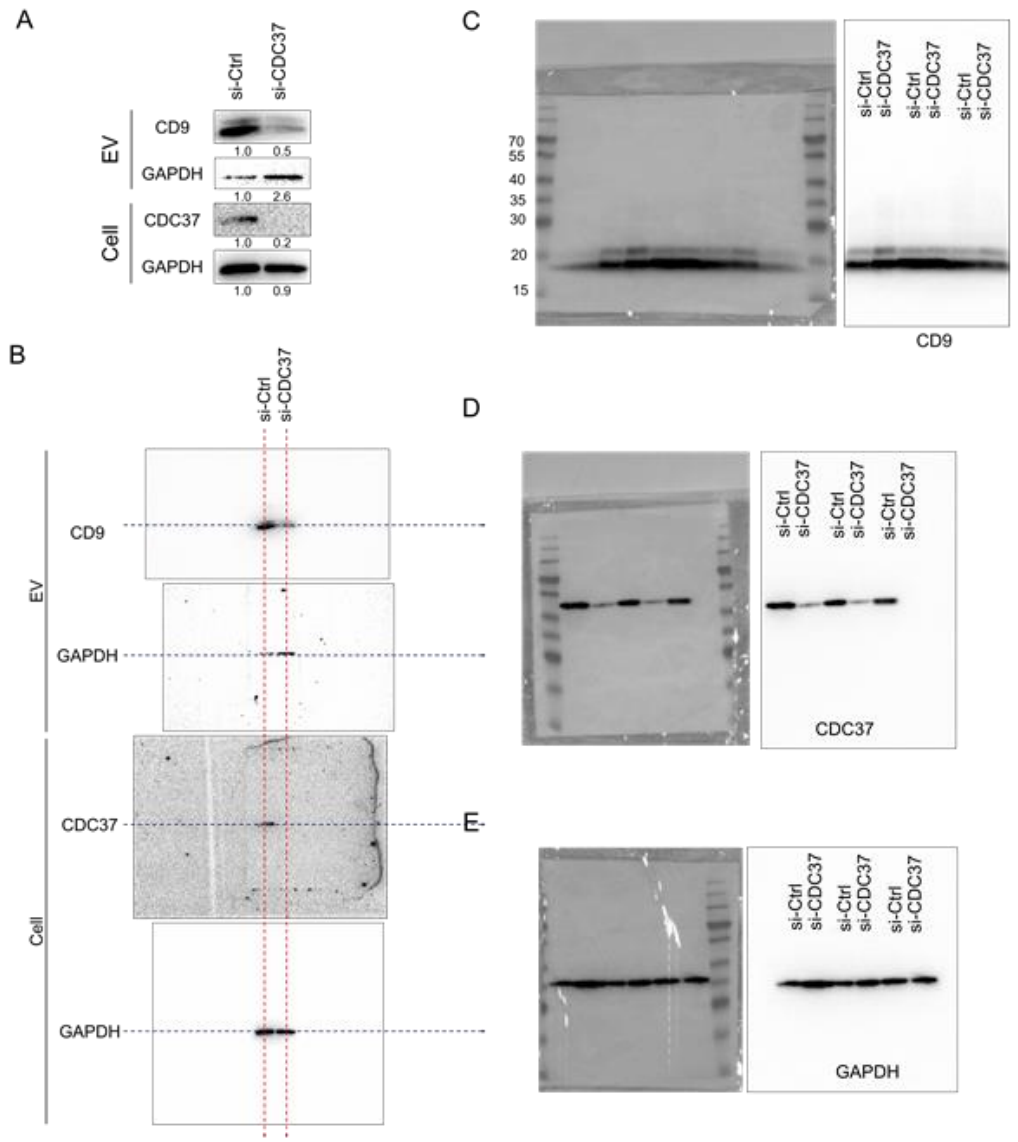

Figure S7. Western blotting showing EV-CD9, EV-GAPDH, Cell-CDC37, and Cell-GAPDH, supporting Figure 5A. (A) Relative intensities of the bands. (B) Full images of the western blotting. For EV-CD9 and -GAPDH, 3 μg each protein samples were applied. For Cell-CDC37 and -GAPDH, 15 μg each protein samples were applied per lane. (C, D, E) Full images of western blotting for (C) CD9, (D) CDC37, and (E) GAPDH in whole cell lysates. Protein samples (15 μg each) were applied per lane.

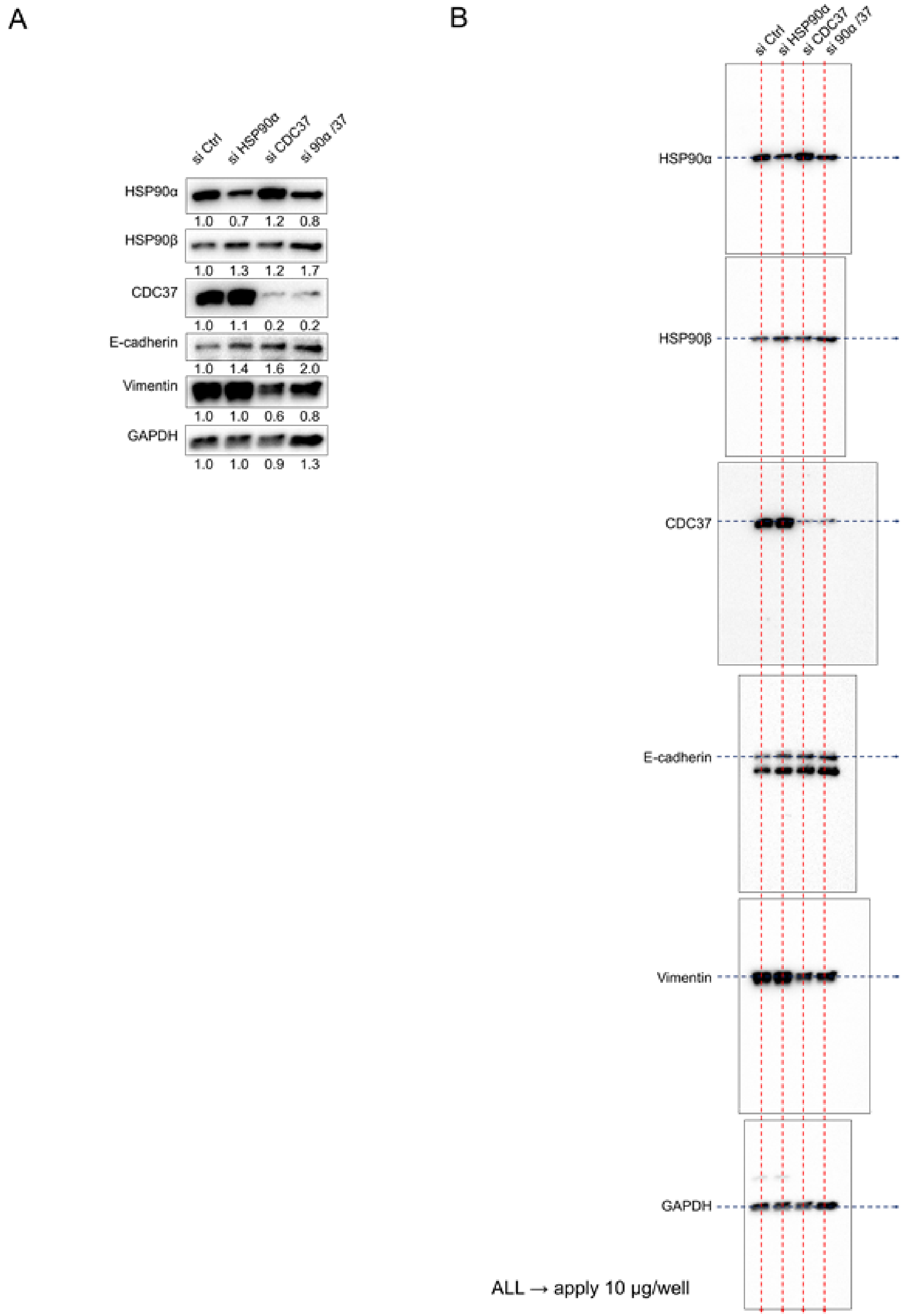

Figure S8. Western blotting showing HSP90α, HSP90β, CDC37, E-cadherin, Vimentin, and GAPDH, supporting Figure 6A. (A) Relative intensities of the bands. (B) Full images of the western blotting. Ten microgram each protein samples were applied per lane .

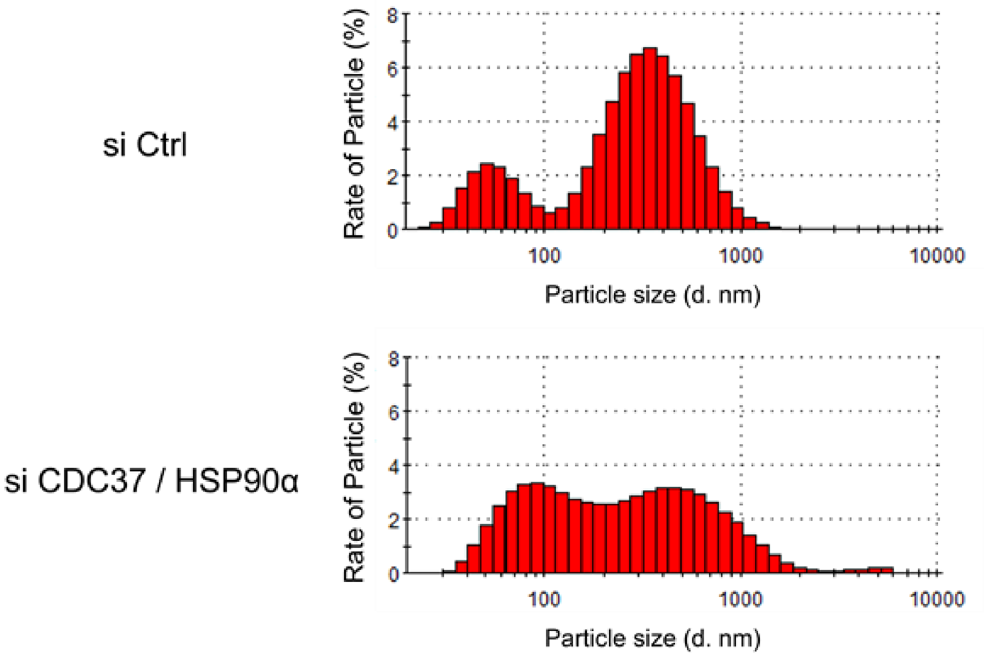

Figure S9. Knockdown of CDC37 and HSP90alpha reduced EV(200-1000nm). PC-3 cells were transfected with siRNA mixture targeting CDC37 and HSP90alpha or non-targeting control siRNA for 24 h. Conditioned medium was passed through a 0.2-micron pore filter and then EV fraction was prepared by polymer-based precipitation method. Particle diameter distribution analysis using Zetasizer.

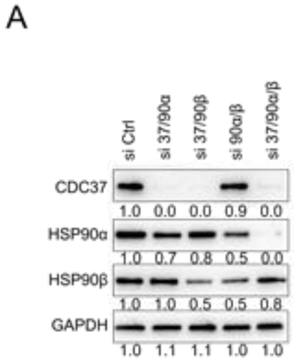

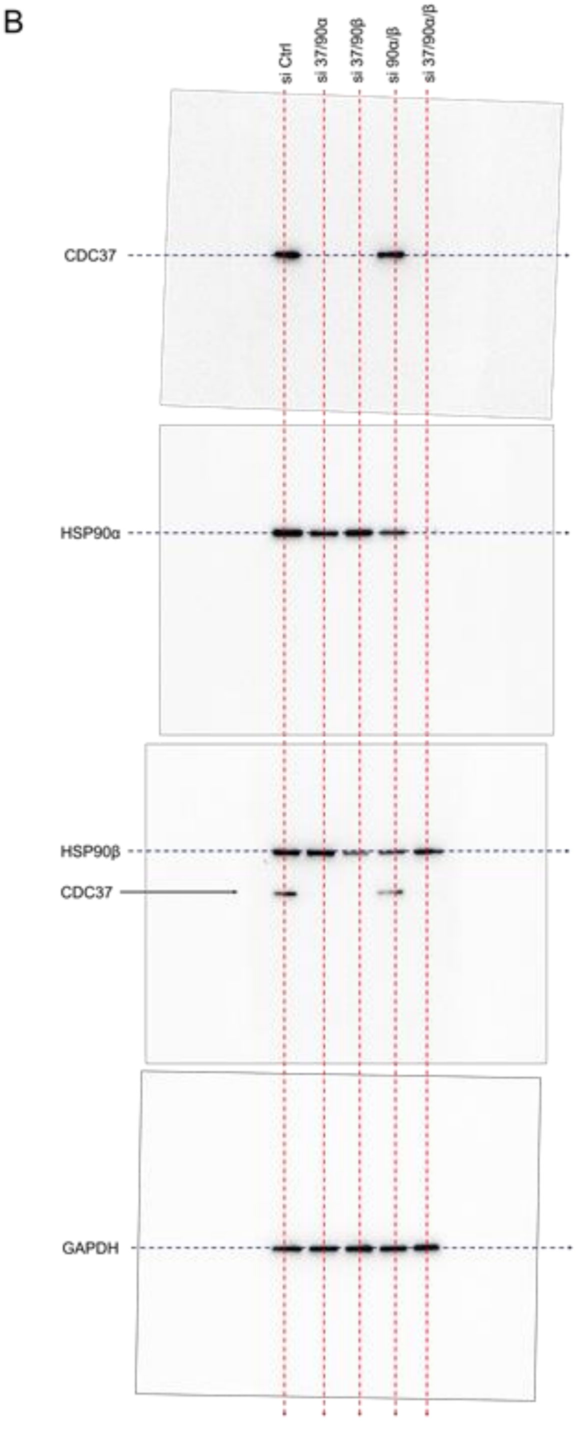

Figure S10. Western blotting showing CDC37, HSP90 α, HSP90 β, and GAPDH, supporting Figure 6C. (A) Relative intensities of the bands. (B) Full images of the western blotting.
